# A Pilot Study on Clinical and Neuroimaging Characteristics of Chinese Posterior Cortical Atrophy: Comparison with Typical Alzheimer’s Disease

**DOI:** 10.1371/journal.pone.0134956

**Published:** 2015-08-12

**Authors:** Xiao-Dan Wang, Hui Lu, Zhihong Shi, Li Cai, Shuai Liu, Shuling Liu, Tong Han, Ying Wang, Yuying Zhou, Xinping Wang, Shuo Gao, Yong Ji

**Affiliations:** 1 Department of Neurology, Tianjin Huanhu Hospital, Tianjin, 300060, China; 2 Department of Radiology, Tianjin Huanhu Hospital, Tianjin, 300060, China; 3 PET-CT Center, General Hospital of Tianjin Medical University, Tianjin, 300052, China; University G. D'Annunzio, ITALY

## Abstract

Posterior cortical atrophy (PCA) is a clinicoradiologic neurodegenerative syndrome characterized by predominant impairment of higher visual functions. Neuroimaging and neuropathological studies show that PCA is probably an atypical presentation of Alzheimer’s disease. However, in China PCA has rarely been studied and remains largely unknown. Our study therefore aimed to analyze the clinical manifestations and patterns of cerebral atrophy, amyloid beta deposition and regional glucose metabolism in Chinese PCA patients, comparing them directly with those of typical Alzheimer’s disease (TAD). Seven PCA patients, 6 TAD patients and 5 controls underwent neuropsychological assessment, MRI scan, ^11^C-PIB PET scan and ^18^F-FDG PET scan. Cerebral atrophy including ventricular enlargement, posterior atrophy and medial temporal lobe atrophy were evaluated with MRI. The uptake of ^11^C-PIB was quantified at the voxel level using the standardized uptake value ratio. Comparisons of regional cerebral glucose metabolism were calculated with statistical parametric mapping. PCA patients showed significant impairment on visuospatial function in neuropsychological assessment. And PCA patients showed more severe posterior atrophy and less severe left medial temporal lobe atrophy compared with TAD patients. The data from ^11^C-PIB PET scanning showed that amyloid beta deposition in PCA was comparable to TAD. Moreover, in PCA the results from ^18^F-FDG PET scanning revealed significant hypometabolism in the temporoparietooccipital region and identified specific hypometabolism in the right occipital lobe, compared with TAD. Our study thus provides a preliminary view of PCA in Chinese patients. A further study with a larger number of subjects would be recommended to confirm these findings.

## Introduction

Posterior cortical atrophy (PCA) is a clinicoradiologic neurodegenerative syndrome characterized clinically by predominant impairment of higher visual functions [1]. PCA was first described by in 1988 by Benson et al. who reported visuo-spatial and visuo-perceptual impairments, alexia, Balint’s syndrome and Gerstmann’s syndrome as the main clinical manifestations of PCA [2]. Memory however is relatively well preserved in the early stages of the disease. Although in rare cases other etiologies have been reported, such as corticobasal degeneration [3, 4], dementia with Lewy bodies [5, 6], and prion disease and subcortical gliosis [7], the most common underlying cause of PCA is Alzheimer’s disease (AD) [8–10].

The diagnosis of PCA depends on demonstrating the core clinical features of the condition, and is further supported by neuroimaging and neuropathological evidence [11]. Studies of regional cerebral glucose metabolism using ^18^F-fluoro-2-deoxy-D-glucose positron emission tomography (^18^F-FDG PET) have shown significant hypometabolism in the occipital, parietal and posterior temporal cortices compared to healthy controls, and in the region of the frontal eye fields as well [12, 13]. In addition, when compared to AD patients, almost exclusively right-sided hypometabolic regions have been reported in PCA patients, extending from the primary visual cortex through the dorsal visual association cortex to the parietal lobe, with maximum reduction in metabolism in the region of the occipito-parietal junction [12]. Moreover, a recent study of PCA patients has shown an area of syndrome-specific hypometabolism in the inferioroccipitotemporal cortex, compared to TAD patients, and in the right lateral temporooccipital cortex, compared to patients with dementia with Lewy bodies [14]. PCA has been reported as showing increased ^11^C-Pittsburgh compound B (^11^C-PiB) uptake, but with no significant difference found in regional or global ^11^C-PIB binding between PCA and TAD, demonstrating that ^11^C-PiB PET may be useful in identifying PCA [15]. Cerebrospinal fluid biomarkers of AD such as protein tau, phosphorylated tau and amyloid beta 1–42 (Aβ1–42) are also recognized as being supportive features in the diagnosis of PCA [16, 17].

Typical Alzheimer’s disease (TAD) is traditionally characterized in terms of episodic memory deficits, with nonamnestic presentations considered as AD variants, one of which is PCA. Although the neuropathology and neuroimaging presentation of PCA has been elaborated on in some studies, mostly of non-Chinese Caucasian populations, the variety of clinical manifestations of this syndrome often leads to delayed diagnosis, particularly in China where its unfamiliarity and lack of recognition has meant that PCA has rarely been reported. In the present study, we decided to address this issue and to analyze the clinical manifestations and pattern of cerebral atrophy, Aβ deposition and regional glucose metabolism in Chinese PCA patients, comparing these directly with TAD patients, using MRI, ^11^C-Pittsburgh compound B PET (^11^C-PiB PET) and ^18^F-FDG-PET.

## Materials and Methods

### Subjects

Subjects were recruited from the cognitive disorder clinic at Tianjin Huanhu Hospital, Tianjin, China, between April 2012 and September 2014 (PCA: n = 7; TAD: n = 6; control: n = 6). Patients were defined as PCA who fulfilled the following criteria [11]: (1) insidious onset and gradual progression; (2) prominent visuoperceptual and visuospatial impairments but no significant impairment of vision itself; (3) relative preservation of memory and insight; (4) evidence of complex visual disorders (e.g., elements of Balint’s syndrome or Gerstmann’s syndrome, visual field defects, visual agnosia, environmental disorientation); (5) absence of stroke, tumor or motor symptoms suggestive of dementia with Lewy bodies. The diagnosis of AD was made according to the criteria of the National Institute of Neurological and Communicative Disorders and Stroke–Alzheimer’s Disease and Related Disorders Associations (NINCDS-ADRDA) for the diagnosis of probable AD [18]. The 5 controls were healthy people without a family history of neurological or psychological disorders who were matched for age with the patients. All subjects underwent neuropsychological assessment by trained neurologists, as well as MRI, ^11^C-PIB PET scan and ^18^F-FDG PET scans. PCA patients and AD patients were matched for age and MMSE score.

### Ethics Statement

Written informed consent was obtained from all subjects and their assigned surrogate decision-makers. This study was approved by the Tianjin Huanhu Hospital Ethics Committee.

### Magnetic resonance imaging

Magnetic resonance images were acquired using a 3.0T SIEMENS Tim Trio MRI scanner. A T1-weighted coronal image was acquired using a three-dimensional spoiled gradient recalled echo inversion recovery prepped sequence (repetition time [TR] = 11 ms, echo time [TE] = 4.94 ms, flip angle [FA] = 20°, 1 mm slice thickness [zero gap], 160 slices, field of view [FOV] = 230 mm × 230 mm). All of the images from the 3T were reconstructed to a size of 256 × 256 with an isotropic resolution of 1×1× 1 mm.

### PET imaging

Head movement was minimized using a polyurethane immobilizer molded around the head. The PET images were acquired on a GE Discovery LS PET/CT scanner in the three-dimensional scanning mode, yielding 35 slices with 4.25 mm thickness that covered the entire brain. ^11^C-PIB PET scans were acquired during 90-min dynamic PET acquisition (34 frames: 4 × 15s, 8 × 30s, 9 × 60s, 2 × 180s, 8 × 300s, 3 × 600s). ^11^C-PIB was administered into an antecubital vein as a bolus injection, with a mean dose of 370–555 MBq. The images were reconstructed to a 128 × 128 matrix (2.5 × 2.5 mm^2^ pixel size).

The ^18^F-FDG study was conducted 1 h after the ^11^C-PIB PET scan using the same scanner, scanning mode, positioning and reconstruction matrix. The subjects received an intravenous injection of 250 MBq ^18^F-FDG and remained in a darkened, quiet room. A 10-min static PET emission scan was performed 60 min after the ^18^F-FDG injection.

### Quantification of ^11^C-PIB uptake

The uptake of ^11^C-PIB was quantified at the voxel level using the region-to-cerebellum ratio, which is identical to the standardized uptake value ratio (SUVR). This simplified quantification enables the utilization of a short 30-min image acquisition.

### Automated region-of-interest analysis

Standardized regions of interest (ROIs) were defined on the MRI template image that represented brain anatomy, in accordance with the Montreal Neurological Institute (MNI). We merged and pooled subsets from the original Automated Anatomic Labeling (AAL) atlas to form the following ROIs: middle frontal gyrus (MFG), medial prefrontal cortex (MPFC), lateral temporal cortex (LTC), hippocampus and parahippocampus (HF+), inferior parietal lobe (IP), posterior cingulate cortex and precuneus (PCCPre), striatum, thalamus, occipital lobe (OL), superior temporal gyrus (STG), and supplementary motor area (SMA).

### ^11^C-PIB PET image analysis

The preprocessing of the ^11^C-PIB imaging data was performed using Statistical Parametric Mapping 8 (SPM8) software and MATLAB 2010b for Windows (Mathworks, Natick, MA, USA). First, ^11^C-PIB integral images (data corrected for radioactive decay summed from 60 to 90 min post-injection) were created from the dynamic PET images (frames 32 to 34) and coregistered to the subject’s MRI images. Second, the magnetic resonance images were segmented into three classes (gray matter, white matter, and cerebrospinal fluid) in SPM8 using 16 non-linear iterations and 7 × 9 × 7 basis functions. Third, the PET images and gray matter magnetic resonance images were normalized using a T1-weighted MRI template that was delivered with SPM to obtain normalization parameters. The application of a 0.5 threshold to the gray matter probability map created a gray matter probability map in the MNI space. The gray matter probability map was then coregistered to the AAL template, and the PET counts were extracted from the gray matter probability map and ROIs. The mean values for all of the regions were calculated from the integral ^11^C-PIB image. Target-to-cerebellum ratios were subsequently calculated for 11 bilateral regions.

### ^18^F-FDG PET image analysis

Spatial preprocessing and statistical analyses of ^18^F-FDG PET images were also performed in all of the subjects using SPM8 software and MATLAB 2010b for Windows. We compared cerebral glucose metabolism in the AD group with that of the control group. We also compared cerebral glucose metabolism between each aMCI subject and the control group. First, ^18^F-FDG PET images were converted to the ANALYZE format and then normalized to the MNI standard proportional stereotaxic space. Second, an isotropic 10 mm full-width half-maximum Gaussian spatial smoothing filter was applied to the image. Third, all of the comparisons of brain metabolism were performed on a voxel-by-voxel basis using a two-sample *t*-test. Statistical significance was determined using an extent threshold of 50 voxels. Regions that reached an uncorrected *P* value of less than 0.001 were considered statistically significant.

### Statistical analysis

Student's t-test was used to compare patients’ characteristics, neuropsychological data and cerebral atrophy between PCA and TAD. Paired Student's t-test was used to compare cerebral atrophy between left side and right side. Two-way ANOVA followed by Bonferroni's post-hoc test was used to analyze the difference of SUVR in brain regions. In all cases, a *P* value of<0.05 was considered significant, unless specified otherwise.

## Results

### Patient characteristics

Detailed patients’ characteristics are showed in Table 1. There was no significant difference between PCA and TAD in age, symptom duration, education, MMSE score, MoCA score and ADL score. And unsurprisingly, the CDT revealed a significant difference between PCA and TAD (*P =* 0.025; PCA < TAD), which indicated impairment of visuospatial function. In this study 6 out of 7 patients with PCA and 1 out of 6 patients with TAD were female; the uneven sex distribution may represent a chance finding, since we are not aware of a bias in favor of women. Furthermore, the most common initial symptoms of patients with PCA were: visual difficulties, inability to find a subject in a room, tendency to lose their way, visual disorientation, and inability to use tools, amongst others. However, the most common initial symptom of patients with TAD was poor episodic memory.

**Table 1:** Patient characteristics.

	PCA	AD	P
**Age (years)**	60.1 ± 2.5	61.0 ± 1.8	0.79
**Gender(female/male)**	6/1	1/5	/
**Symptom duration (years)**	2.4 ± 0.5	2.5 ± 0.4	0.92
**Education (years)**	10.6 ± 0.6	9.5 ± 1.4	0.48
**MMSE score**	15.0 ± 2.0	15.0 ± 2.8	1.00
**MoCA score**	6.4 ± 1.4	10.0 ± 2.8	0.25
**ADL score**	37.0 ± 5.5	28.7 ± 3.0	0.23
**CDT score**	0.6 ± 0.2	1.7 ± 0.5	**0.025**

Data are the mean ± SEM (except gender). Significant results are marked in bold (Student's t-test).

### Cerebral Atrophy Analysis with MRI

In this study, all patients underwent MRI scan of brain, and the atrophy of our area of interest was assessed according to Korf et al. for medial temporal lobe atrophy (MTA), O’Donovan et al. for ventricular enlargement (VE) and Koedom et al. for posterior atrophy (PA) [19–21]. The data showed that the left MTA of TAD was significantly more obvious than in that of PCA, while there was no difference in either the right MTA or VE between the PCA group and the TAD group. As expected, the severity of PA was significantly higher in the PCA group than in the TAD group (Table 2). Moreover, in all three positions accessed in the present study, the atrophy was symmetric (*P*>0.05).

**Table 2:** Cerebral atrophy analysis with MRI.

		PCA	tAD	P
**MTA**	**L**	1.4 ± 0.4	2.8 ± 0.4	**0.026**
	**R**	1.4 ± 0.5	2.5 ± 0.4	0.15
**VE**	**L**	1.9 ± 0.1	1.8 ± 0.3	0.94
	**R**	1.9 ± 0.1	1.7 ± 0.2	0.46
**PA**	**L**	2.1 ± 0.4	0.5 ± 0.2	**0.0061**
	**R**	2.3 ± 0.3	0.5 ± 0.2	**0.0006**

All patients underwent MRI scan (PCA: n = 7; TAD: n = 6). P values are PCA vs TAD, Significant results are marked in bold (Student's t-test). (MTA = Medial temporal lobe atrophy; VE = Ventricular enlargement; PA = Posterior atrophy).

### Aβ deposition analysis with ^11^C-PiB PET

**Fig 1:**
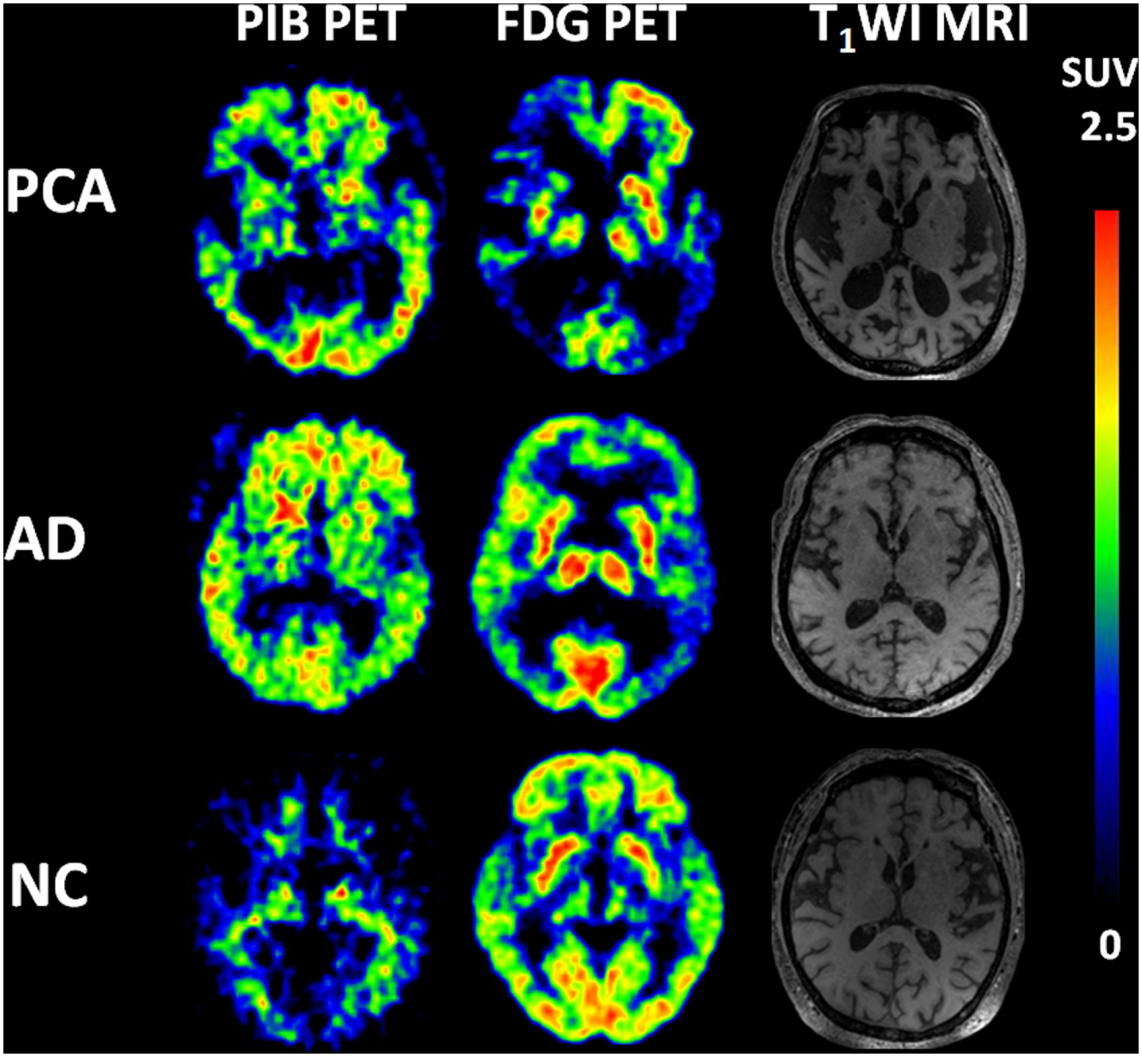
PiB (left), FDG (right) and MRI images in PCA and TAD patients. PiB and FDG images are quantified by SUVR with the displayed color scales. In both PCA and TAD patients, the clearance rate of radioactivity was slower symmetrically or asymmetrically in the cortex of frontal lobe, parietal lobe, lateral temperal lobe, praecuneus, posterior cingulate and occipital lobe.

**Fig 2:**
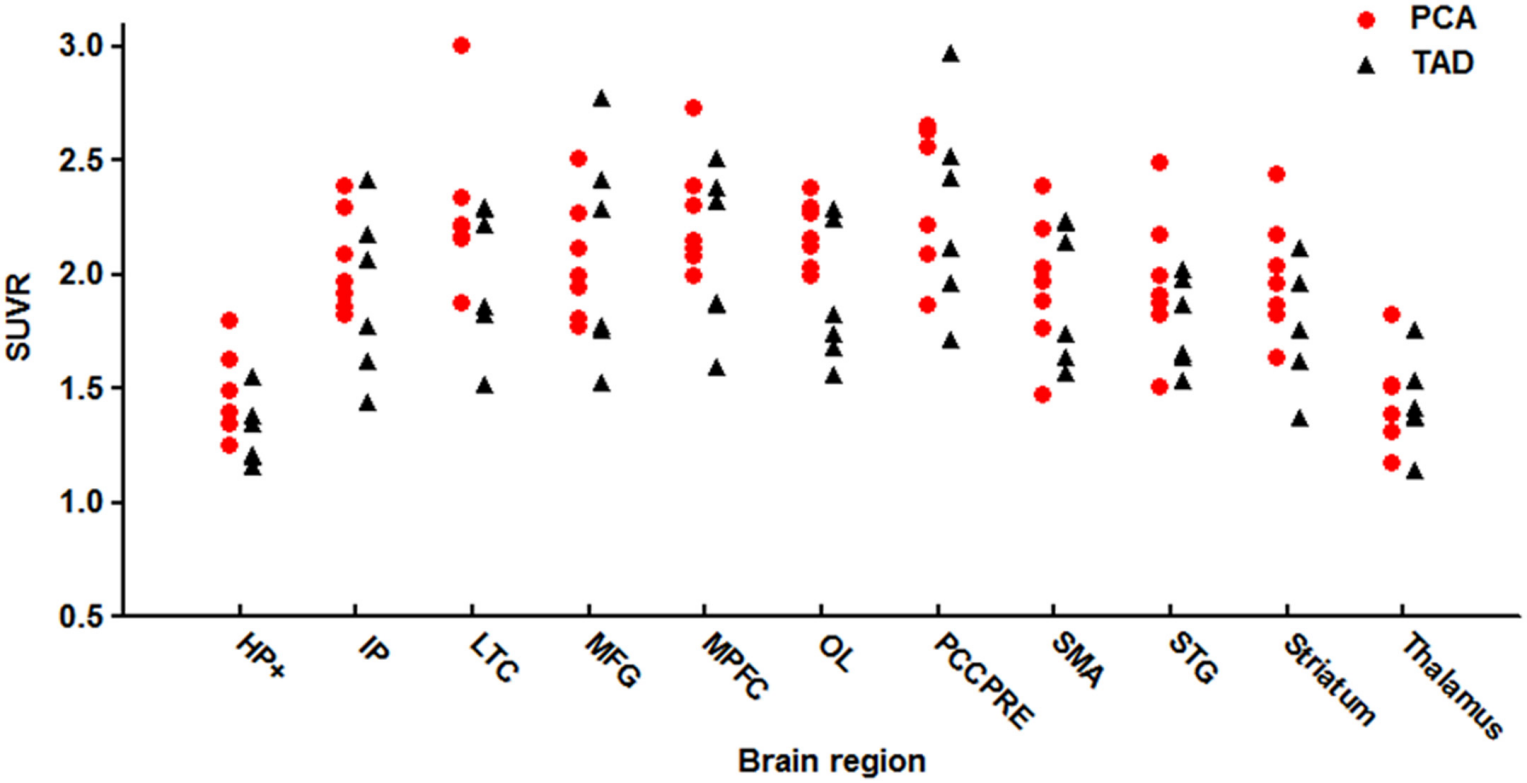
PIB SUVR in anatomical regions of PCA patients (circles) and TAD patients (triangles). Standardized regions of interest (ROIs) were defined on the MRI template image that represented brain anatomy in accordance with the Montreal Neurological Institute (MNI). We merged and pooled subsets from the original Automated Anatomic Labeling (AAL) atlas to form the following ROIs: middle frontal gyrus (MFG), medial prefrontal cortex (MPFC), lateral temporal cortex (LTC), hippocampus and parahippocampus (HF+), inferior parietal lobe (IP), posterior cingulate cortex and precuneus (PCCPre), striatum, thalamus, occipital lobe (OL), superior temporal gyrus (STG), and supplementary motor area (SMA).

The presence of cortical Aβ deposition was confirmed by ^11^C-PiB PET in all cases. After 45 min of PIB injection, visual analysis showed that the clearance rate of radioactivity was slower symmetrically or asymmetrically in the cortex of the frontal lobe, parietal lobe, lateral temperal lobe, praecuneus, posterior cingulate and occipital lobe in both PCA and TAD (Fig 1). Voxel-based automatic quantitative analysis was used to determine the mean PIB SUVR of regions of interest. The data showed both PCA and TAD were PIB positive [22]. In addition, the SUVR of PCA was significantly higher than in controls in hippocampus and parahippocampal gyrus, inferior parietal lobe, lateral temperal lobe, middle frontal gyrus, medial prefrontal cortex, occipital lobe, posterior cingulate/praecuneus, supplementary motor area, superior temporal gyrus and striatum; this was also the case in TAD. We did not find a significant difference of regional Aβ deposition between PCA and TAD (Fig 2).

### Cerebral glucose metabolism analysis with ^18^F-FDG PET: comparison of PCA with TAD

**Fig 3:**
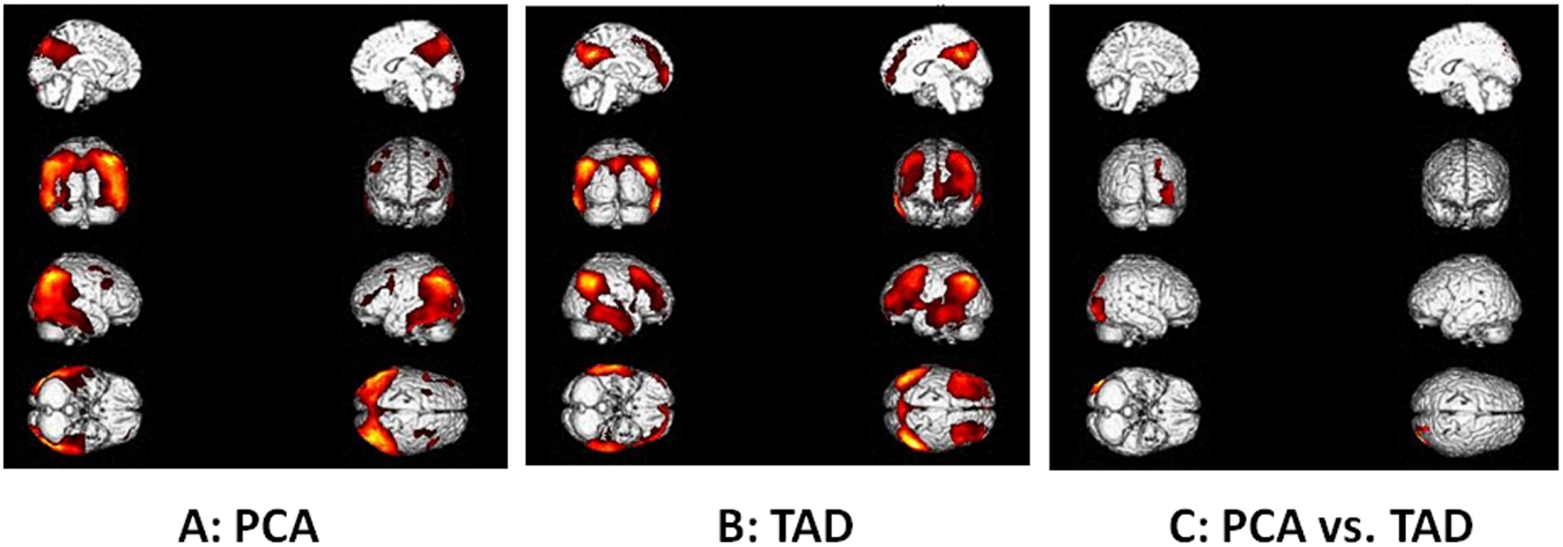
Topography of hypometabolism in PCA patients (A) and TAD patients (B). Syndrome-specific area of hypometabolism in PCA vs TAD (C). (see details in Materials and Methods).

In the present study, we analyzed cerebral glucose metabolism with ^18^F-FDG PET of both PCA patients and TAD patients. SPM analyses revealed significant hypometabolism in the right praecuneus (BA7, 19), left praecuneus (BA7, 39), inferior parietal lobule (BA40), superior temporal gyrus (BA22), middle temporal gyrus and left angular convolution (BA39, 37), right supramaginal gyrus (BA40), superior occipital gyrus (BA19), right occipital gyrus (BA18, 37), left occipital lobule (BA18) and fusiform gyrus (BA19, 20) in PCA (Fig 3A). In TAD, significant hypometabolism was found in the left middle frontal gyrus (BA9, 46, 6), left inferior frontal gyrus (BA10, 20, 8), left medial frontal gyrus (BA10), left precentral gyrus (BA9), right praecuneus (BA40, 31, 39), middle temporal gyrus (BA21), inferior temporal gyrus (20), left angular convolution (BA39), supramaginal gyrus (BA40), fusiform gyrus (BA20), left insular lobe (BA13) and left anterior cingulated (BA32) (Fig 3B). Comparing PCA with TAD, there were significant differences between the two groups: PCA patients showed specific clusters of hypometabolism in the right occipital lobe (BA18, 19, 37) (Fig 3C).

## Discussion

The present study explored for the first time the clinical manifestations, cerebral atrophy, cerebral Aβ deposition and glucose metabolism of Chinese PCA patients, and compared them with those of TAD patients. The PCA group showed similar levels of performance on non-visuospatial cognitive tasks compared to the TAD group, but showed marked impairment on visuospatial tasks. Moreover, PCA patients showed more PA and less left MTA compared with TAD patients. In this study, the data from ^11^C-PiB PET showed that Aβ deposition in PCA was comparable to TAD, and indicated that PCA was of the same pathology as TAD. On the other hand, the ^18^F-FDG PET results revealed significant hypometabolism in the temporoparietooccipital region in PCA patients and identified specific hypometabolism in the right occipital lobe compared with TAD. Our study therefore provided a preliminary view with MRI, ^11^C-PiB PET and ^18^F-FDG PET of PCA in Chinese patients and will play an important role in identifying PCA patients in China.

Though PCA patients have been reported as being younger than TAD patients [1], we did not see this tendency in the present study, as the PCA group and TAD group were both matched for age and MMSE score. However, the age of all the subjects was around 60 years which indicated that the onset age of PCA was indeed younger than that of TAD. Moreover, we also found no significant difference in education, MoCA score or ADL score between PCA and TAD. Notably, patients with PCA performed significantly worse than the TAD patients in the CDT, suggesting visuospatial function impairment. Combined with the initial symptoms of patients, our results confirm that PCA in Chinese patients showed similar characteristics to those in the literature [23–26].

In line with previous studies which showed occipitoparietal atrophy on MRI, predominant posterior cortical atrophy was found in MRIs of PCA patients when compared to TAD patients in our study. The data from MRIs also demonstrated less severity of MTA in PCA patients than in TAD patients. Posterior cortical atrophy without medial temporal atrophy is classically described in MRI findings of PCA with the atrophy progressively worsening over time. One study with repeated MRI showed that cortical atrophy affected first the superior parietal and inferior temporal cortices, then the inferior parietal and occipital areas one year later, then the medial temporal lobe another year later, and ultimately global cortices [27]. Other studies have reported that PCA patients exhibited significantly more atrophy in the right parietal, bilateral posterior parietal regions and the occipital lobe, whereas TAD patients showed greater atrophy in the hippocampi and the left medial temporal lobe [28–30]. These findings will help to differentiate PCA from TAD at the early stage of the disease. The MRI findings in the present study were comparable to those of previous studies. Moreover, the results were also consistent with the clinical manifestations: PCA patients showed higher levels of impairment in visuospatial and visuoperceptual functions, and TAD patients presented with greater impairments in memory. These findings support an association between neural atrophy and the cognitive deficits observed in PCA and TAD.

According to the literature, the etiologies of PCA include AD, dementia with Lewy bodies, subcortical gliosis, corticobasal degeneration, and prion associated diseases (see intro). AD is the most frequent cause of PCA, accounting for about 80% of cases [6, 7, 24]. In our study, we analyzed the presence of cortical Aβ deposition with ^11^C-PiB PET. Consist with the findings of the previous study [15], our data showed no difference in regional Aβ deposition between PCA and TAD, which indicated that the pathology of PCA in Chinese patients was probably AD. However, studies have shown the pattern of the distribution of plaques and neurofibrillary tangles in PCA patients to be inconsistent. Some studies have demonstrated similar plaque distribution between PCA and typical AD [6, 24], whereas other researchers have demonstrated differences in both plaques and neurofibrillary tangles [31–33]. Studies assessing CSF biomarkers (Aβ1–42, total tau and phosphorylated) have reported similar findings in PCA compared with TAD [15–17, 34, 35], supporting the conclusion that PCA is associated with AD pathology.

We found extensive hypometabolism of the bilateral posterior cortex, most prominently in the parietooccipital regions and temporooccipital regions in PCA, which is in agreement with previous studies [12, 13, 26]. Hypometabolism extended from the primary visual cortex through the dorsal visual association cortex to the parietal lobe, suggesting disruption to the visuospatial function, and through the ventral visual association cortex to the temporal lobe, suggesting disruption to the visuoperceptive function. In this study, we found no hemispherical asymmetry of hypometabolism, which is inconsistent with the previously reported right hemispheric dominance of metabolic changes in PCA [12, 36]. The hypometabolism in TAD was found in the frontal lobe, occipitoparietal area and cingulate gyrus. Comparable abnormalities of the inferior and lateral occipitotemporal cortex have been frequently reported in studies examining TAD patients [37, 38]. Our study found a significant decrease in cerebral glucose metabolism exclusively in the right occipital cortex of PCA patients compared with TAD, while a previous study had shown specific hypometabolism in PCA patients in the right lateral temporooccipital cortex compared to TAD [14]. One explanation for the difference between our study and the previous one might be the relative late stage of disease in our study. An observed positive correlation of parietooccipital hypoperfusion and disease duration has been reported, suggesting that in the course of the disease the decline of functional brain networks might be progressive [26]. Our data also correlated with the pathology study, which showed that patients with PCA have a higher density of neurofibrillary tangles in the occipital regions [9, 24, 33]. Our results as well as the previous findings thus lead to the assumption that a syndrome-specific disruption of networks may help to distinguish PCA from TAD.

PCA has previously been reported as accounting for 5% of all cases with suspected AD [39] and approximately 4% of all new dementia cases (Croisile B, 2004). In China, the number of AD patients exceeded 10 million by the year 2010 and the incidence of dementia is 9.87% of people aged over 65 [40]. Therefore there are probably more than 500,000 PCA patients in China and the number is increasing rapidly. However, in Chinese studies, the neuropathology and neuroimaging of PCA remains almost wholly unreported on. This may be because investigations for the purposes of research and diagnosis of PCA are very expensive, thus limiting the research and resulting in substantial underdiagnosis of PCA in China. Since PCA in the Chinese population is barely known and hardly studied at all, the present study was therefore undertaken to address this deficit, using ^11^C-PiB PET and ^18^F-FDG PET.

One limitation of this pilot study is the low number of subjects included. Therefore, the results should be interpreted with caution. Another limitation is the relatively few male patients in the PCA group, this point should be addressed in future studies. However, to the best of our knowledge, our study was the first attempt to explore the clinical and neuroimaging characteristics of Chinese PCA. Thus, we consider our findings will provide the basis for future study. To confirm our results, a further study with a larger number of subjects will be needed.

In conclusion, our study elaborated clinical manifestations and patterns of cerebral atrophy, Aβ deposition and regional glucose metabolism in Chinese PCA patients. Compared with TAD, the PCA group showed marked impairment on visuospatial function, more severity of PA and specific hypometabolism in the right occipital lobe. However, cerebral Aβ deposition in PCA was comparable to TAD. Our findings provided a preliminary view of PCA in Chinese patients and need to be confirmed with larger sample size.
